# Forgotten People, Forgotten Diseases: The Neglected Tropical Diseases and Their Impact on Global Health and Development

**DOI:** 10.3201/eid1503.081597

**Published:** 2009-03

**Authors:** C. Ben Beard

**Affiliations:** Centers for Disease Control and Prevention, Fort Collins, Colorado, USA

**Keywords:** Tropical diseases, schistosomiasis, filariasis, mycobacteria, trypanosomiasis, leishmaniasis, dengue, leptospirosis, rabies, book review

Forgotten People, Forgotten Diseases is an interesting and highly informative book about the global status of neglected tropical diseases (NTDs). Author Peter Hotez introduces NTDs by describing them in general, their historical importance and global impact, and their shared characteristics. According to Hotez, NTDs are among the most common infections from antiquity and occur in the world’s poorest people. Their distribution and health and economic effects are similar to those of AIDS, malaria, and tuberculosis. NTDs, however, are much less well known than these diseases and frequently display high rates of illness but few deaths, promote poverty, and create profound social stigma.

Twelve well-illustrated chapters address the important NTDs, including soil-transmitted helminth infections, schistosomiasis, filariasis, onchocerciasis, trachoma, mycobacterial infections, trypanosomiasis, leishmaniasis, dengue, leptospirosis, and rabies. Hotez discusses what these diseases are, where they occur, and who they affect. The final chapters focus on prospects to prevent and control NTDs and the need for additional advocacy. Hotez emphasizes the need for new safer and more effective drugs, as well as for so-called “anti-poverty vaccines,” which by promoting health will open doors to economic advancement and stability, goals that have been all but impossible in developing countries, largely because of NTDs.

Few people are more qualified to write such a book than Hotez, president of the Sabin Vaccine Institute and a pioneer in hookworm molecular genetics, physiology, immunology, and pathogenesis. This easy-to-read and up-to-date text undoubtedly will prove useful to graduate students, volunteers, advocates, healthcare professionals, and others interested in global health and equality. Forgotten People, Forgotten Diseases is an essential read for every serious student of tropical medicine and global infectious diseases.

